# Embedding a feruloyl esterase active site into a thermophilic endoxylanase scaffold for the degradation of feruloylated xylans

**DOI:** 10.1016/j.csbj.2025.09.003

**Published:** 2025-09-03

**Authors:** Rubén Muñoz-Tafalla, Isabel Cea-Rama, Fadia V. Cervantes, Jose L. Gonzalez-Alfonso, Francisco J. Plou, Julio Polaina, Julia Sanz-Aparicio, Manuel Ferrer, Víctor Guallar, David Talens-Perales

**Affiliations:** a Department of Life Sciences, Barcelona Supercomputing Center (BSC), Barcelona 08034, Spain; b PhD program in Biotechnology, University of Barcelona (UB), Barcelona 08028, Spain; c Institute of Physical Chemistry Blas Cabrera (IQF), CSIC, Madrid 28006, Spain; d Instituto de Catalisis y Petroleoquimica (ICP), CSIC, Madrid 28049, Spain; e Institute of Agrochemistry and Food Technology, Spanish National Research Council (IATA-CSIC), Paterna, Valencia 46980, Spain; f Institució Catalana de Recerca i Estudis Avançats (ICREA), Barcelona 08010, Spain

**Keywords:** Feruloyl esterase, Protein Engineering, PluriZyme, Xylan, Xylanase

## Abstract

The structural complexity of xylan makes its complete degradation challenging. Strategies to improve its hydrolysis often requires enzyme cocktails with multiple specific activities or proteins harboring multiple catalytic domains. Here, we introduce a novel approach through the design of Xyn11_m1_, a multifunctional enzyme that combines endoxylanase and feruloyl esterase activities, two catalytic functions involved in the hydrolysis of feruloylated xylans. Using the PluriZyme concept, an artificial feruloyl esterase active site was engineered into the scaffold of a thermophilic glycoside hydrolase family 10 xylanase, Xyn11, from *Pseudothermotoga thermarum*. Computational design, guided by protein energy landscape exploration simulations, revealed a surface cavity that could accommodate feruloyl-L-arabinose and a xylopentaose (a 5-xylose xylan polymer) bearing a single feruloyl-L-arabinose substitution on the central xylose unit. This cavity was subsequently remodeled into a serine–histidine–aspartic/glutamic acid catalytic triad with feruloyl esterase activity. Molecular dynamics simulations confirmed the stability of the engineered active site. Xyn11_m1_ was successfully produced, crystallized, and characterized, and its xylanase activity at 90 °C against oat spelt xylan was comparable to that of the wild-type enzyme (713 ± 4 vs. 600 ± 8 units/mg), and it also displayed feruloyl esterase activity against methyl ferulate (140 ± 5 units/mg), a capability lacking in Xyn11. Notably, Xyn11_m1_ exhibited approximately 2.5-fold greater activity compared with Xyn11 (513 ± 27 vs. 222 ± 9 units/mg) against wheat bran xylan containing ferulic acid ester linked to arabinofuranosyl residues. This dual functionality enables efficient degradation of feruloylated xylans, highlighting the potential of PluriZymes to advance biomass deconstruction technologies.

## Highlights

• PluriZyme design added a Ser–His–Glu triad to a thermostable xylanase scaffold
• A scaffold combining endoxylanase and feruloyl esterase activities was designed
• The new scaffold retained xylanase activity on oat spelt xylan at 90ºC
• Feruloyl esterase incorporation gave 2.5-fold higher activity on wheat bran xylan
• Multifunctional PluriZymes streamline deconstruction of feruloylated xylans

## Graphical abstract

**Figure:**
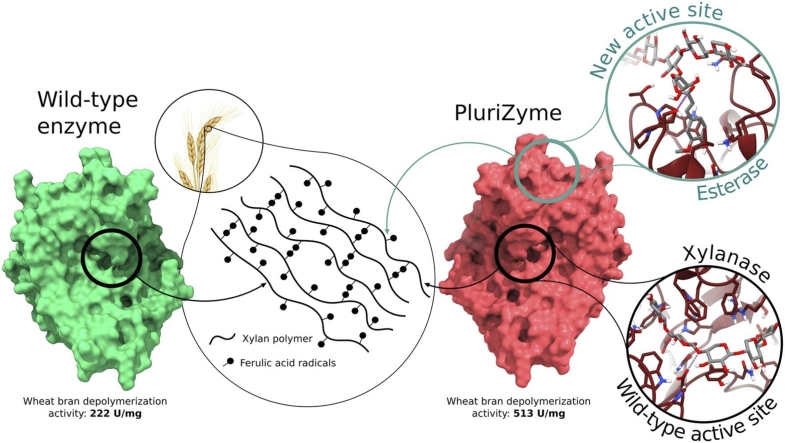
Comparative schematic representation of wild-type versus engineered PluriZyme activity on wheat bran. A magnified view of wheat bran showing a polymeric arabinoxylan structure with feruloylated arabinofuranose substitutions and dimeric crosslinks. On the left, the surface of the wild-type enzyme (green) is shown with its native xylanase active site circled in black. This enzyme has an activity of 222 ± 9 units/mg of wheat bran depolymerization. On the right, the engineered PluriZyme (red) displays two active sites, the native xylanase (black circle) and the newly introduced feruloyl esterase active site (blue circle), together achieving 513 ± 27 units/mg depolymerization activity. The arrows indicate cleavage sites on the xylan backbone and at the ferulic acid ester linkages.

## 1 Introduction

Xylan is an essential structural component of the plant cell wall. It is the second most abundant polysaccharide in plant biomass after cellulose [1]. Structurally, xylan comprises a linear backbone of β-1,4-linked xylose units, which display different types of chemical modifications. These modifications follow characteristic patterns in different groups of plants. Thus, in dicots, the backbone xylosyl residues are frequently acetylated and substituted with glucoronosyl and methyl-glucoronosyl residues. In monocots, xylosyl residues are very frequently substituted with arabinosyl residues, which in turn are frequently esterified with hydroxycinnamates, ferulate or para-coumarate, yielding a more complex structure [1], [2]. This complexity is further increased because feruloyl groups can dimerize, resulting in inter- and intra-cross-linkage of xylan chains, and can also be linked to lignin and proteins [3]. Xylan abundance in plant material is highly important from a biotechnological perspective. Products of xylan hydrolysis can be converted into biofuels or a wide variety of valuable compounds [4], [5]. In some industrial processes, such as paper production, xylan is considered an undesirable component that must be removed to achieve efficient pulp bleaching. In this context, xylan-degrading enzymes offer an efficient, environmentally friendly alternative to harsh, chlorine-based conventional procedures [6]. They are also increasingly valued for their ability to degrade xylan into prebiotic xylooligosaccharides [7], [8]. However, efficient enzymatic hydrolysis of xylan requires the cooperative action of multiple enzymes, particularly when dealing with more complex forms of xylan, such as those found in cereals [2]. Glycoside hydrolases cleave the glycosidic bonds between successive xylose residues and remove arabinosyl and glucuronoyl side chains, whereas esterases hydrolyze acetyl and feruloyl ester linkages [9].

In this work, we report a novel approach for the enzymatic hydrolysis of xylan, particularly feruloylated xylan, through the design and construction of an enzyme that combines two activities, namely, endoxylanase and feruloyl esterase. These two representative classes of enzymes are involved in the hydrolysis or modification of polysaccharides and oligosaccharides, as cataloged in the CAZy database [10], and both are required for effective degradation. The PluriZyme concept [11], more precisely, the capacity to introduce artificial hydrolytic active sites by transforming substrate binding sites into catalytic sites, motivated this study. This approach has been successfully applied to assemble a Ser–His–Asp catalytic triad that supports ester hydrolysis in multiple unrelated protein scaffolds [12], [13], [14], introducing the possibility of combining different biochemistries within a single enzymatic framework, for example, esterase–protease, transaminase–esterase, oxidase–esterase, or even Friedel–Crafts alkylation–esterase activities. These possibilities were explored by starting with an endoxylanase scaffold, namely, Xyn11 from *Pseudothermotoga thermarum* [15], and searching for a binding pocket with favorable binding energies for target feruloylated substrates, followed by the incorporation of an artificial Ser–His–Asp/Glu catalytic triad that remains in a catalytically active conformation.

Our aim was not to create an endoxylanase that outperforms previously reported native or engineered variants. Rather, this proof-of-concept study sought to implement and evaluate the suitability of the PluriZyme approach to introduce artificial feruloyl esterase activity into a native thermophilic endoxylanase scaffold. By doing so, we aimed to expand the catalytic potential of the original enzyme, enabling it not only to hydrolyze xylan but also to efficiently degrade feruloylated xylans.

## 2 Materials and methods

### 2.1 Protein and ligand preparation for in silico analysis

The xylanase studied in this work, named Xyn11, was originally cloned as a synthetic gene encoding an enzyme sequence from *Pseudothermotoga thermarum* (PDB: 7NL2). Xyn11 is a globular single-domain protein formed by 342 amino acids. It was solved at 1.8 Å resolution using X-ray crystallography. The protein folds into a (β/α)_8_ barrel (TIM-barrel) architecture, which is typical of glycoside hydrolase family 10 (GH10) xylanases [15]. The protein structure of Xyn11 was prepared using the PrepWizard module from the Maestro suite [16]. This software predicts the amino acid protonation states and orientations of potential hydrogen bonds according to the optimal operating pH of the enzyme, which is 7.0. The ligands used were feruloyl-L-arabinose for the initial explorations, followed by a 5-unit xylan polymer with feruloyl-L-arabinose bound to the central subunit. In this model, α-L-arabinofuranose is α-(1→3)-linked to the central β-D-xylopyranose unit of the xylan backbone at its O-3 position, and the ferulic acid moiety is esterified to the O-5 position of arabinose, which is consistent with the known substitution pattern of arabinoxylans. Both substrates were modeled using the OPLS2005 force field [17] (Supplementary Fig. 1 A).

### 2.2 Protein Energy Landscape Exploration (PELE)

PELE was used to map the interaction energy landscape between the ligand and the protein surface. PELE is a Monte Carlo-based algorithm coupled with normal mode, side chain and minimization prediction methods [18]. This method starts with the sampling of different microstates by applying perturbations in the translation and rotation of the ligand. Afterward, the flexibility of the protein is accounted for by applying normal modes through the anisotropic network model (ANM) approach. Once the system has been perturbed, all side chains of the residues that are near the ligand are sampled with an experimental library of rotamers to avoid steric clashes. Finally, a truncated Newton minimization with the OPLS2005 force field [17] is performed, and the new microstate is accepted or rejected according to the metropolis criterion.

A detailed description of how to design a PluriZyme has been provided recently [11]. Briefly, the design first involves finding a substrate binding site using a global PELE search [19], followed by a mutation generation step coupled with an induced fit procedure and molecular dynamics (MD) refinement. The new active site must be well organized, and all distances and angles must be appropriate.

### 2.3 Molecular dynamics (MD)

To ensure the stability of the interactions, MD simulations of the selected mutants were performed. MD were run with AMBER99 [20], with simulations lasting a total of 200 ns, during which the results of the state of the system were obtained every 100 ps. Each system was explicitly solvated in a TIP3P water box and neutralized with counterions (Na⁺ or Cl⁻), ensuring overall charge neutrality. For each system, we generated 8 trajectories, amounting to a total time of 1.6 μs. This provides substantial sampling of the conformational space and is sufficient to observe relevant ligand–enzyme interactions. The temperature at which the simulations were run was 300 K. Prior to production runs, the systems underwent energy minimization, followed by equilibration in two phases: 400 ps under an NVT ensemble and 1 ns under an NPT ensemble to stabilize density. All the simulations were performed under periodic boundary conditions.

### 2.4 Crystallization and X-ray structure determination of Xyn11_m1_

Initial crystallization conditions were explored using high-throughput techniques with a NanoDrop robot (Innovadyne Technologies, CA, USA) using 5 mg/mL protein in Tris (20 mM, pH 7) and NaCl (50 mM), protein reservoir ratios of 1:1 and commercial screens, including Index (Hampton Research, CA, USA), JBScreen JCSG and JBScreen PACT (Jena Bioscience, Jena, Germany). After three days, bar-shaped crystals were grown in 20 % PEG3350, 0.1 M Bis-Tris-propane (pH 8.5), and 0.2 M sodium formate in drops made of 250 nL of protein and 250 nL of reservoir. For data collection, the crystals were transferred to a cryoprotectant solution consisting of mother liquor and glycerol (25 % (v/v)) and then cooled in liquid nitrogen. Diffraction data were collected using synchrotron radiation on the XALOC beamline at ALBA (Cerdanyola del Vallés, Spain). Diffraction images were processed with XDS [21] and merged using AIMLESS from the CCP4 package [22]. The crystal was indexed to the P2_1_2_1_2_1_ space group, with two molecules in the asymmetric unit and a 51 % solvent content within the unit cell. The data collection statistics are given in Table 1. The structure of Xyn11_m1_ was solved with difference Fourier synthesis [23] using the coordinates from Xyn11 as a template (PDB Code 7NL2). Crystallographic refinement was performed using the program REFMAC [24] within the CCP4 suite, with automatic local noncrystallographic symmetry (NCS). The free *R* factor was calculated using a 5 % subset of randomly selected structure factor amplitudes that were excluded from automated refinement. Subsequently, IPT and glycerol molecules were manually built into the electron density maps with Coot8 [25], and water molecules were included in the model, which, combined with more rounds of restrained refinement, reached the *R* factors listed in Table 1. The figures were generated with PyMOL. The crystallographic statistics of Xyn11_m1_ are listed in Table 1.

**Table 1:** Crystallographic statistics of Xyn11_m1_.

Values in brackets refer to the high-resolution shell	
**Crystal data**	Xyn11_m1_
Space group	P2_1_2_1_2_1_
Unit cell parameters	
a (Å)	90.32
b (Å)	95.55
c (Å)	101.34
**Data collection**	
Beamline	XALOC(ALBA)
Temperature (K)	100
Wavelength (Å)	0.979185
Resolution (Å)	2.1 (2.06–2.1)
**Data processing**	
Total reflections	258521 (21236)
Unique reflections	51806 (4197)
Multiplicity	5.0 (5.1)
Completeness (%)	99.9 (99.9)
Mean *I*/σ (*I*)	15.8 (2.6)
*R*_*merge*_^*†*^ (%)	6.4 (59.3)
*R*_*p*__im _^*††*^ (%)	3.1 (29.0)
Molecules per ASU	2
**Refinement**	
R_work_/R_free_^*†††*^ (%)	16.1/19.6
**N° of atoms/average B** (Å^2^)	5976/37.73
Macromolecule	5635/37.14
Ligands	66/74.39
Solvent	275/41.14
**Ramachandran plot** (%)	
Favored	97.9
Outliers	0.3
**RMS deviations**	
Bonds (Å)	0.009
Angles (°)	1.469
PDB accession code	8BBI

^†^Rmerge = ∑hkl ∑I | Ii(hkl) – [I(hkl)]|/∑hkl ∑I Ii(hkl), where Ii(hkl) is the ith measurement of reflection hkl and [I(hkl)] is the weighted mean of all the measurements.

^††^Rpim = ∑hkl [1/(N – 1)] ½ ∑I | Ii(hkl) – [I(hkl)]|/∑hkl ∑I Ii(hkl), where N is the redundancy for reflection hkl.

^†††^Rwork/Rfree = ∑hkl | Fo – Fc |/∑hkl | Fo |, where Fc is the calculated and Fo is the observed structure factor amplitude of reflection hkl for the working/free (5 %) set.

### 2.5 Docking simulations

The crystal structure of Xyn11_m1_ was used as a template for the docking experiments. All resolved water molecules and other ligands were removed from the structure prior to protein preparation and subsequent docking simulations. The coordinates of ethyl 4′-hydroxy-3′-methoxycinnamate, an ethyl derivative of ferulic acid, were obtained from the PDB deposition website with the PDB code 3PFB. Substrate docking was performed using AutoDock Vina [26], with Ser271, Glu272, His275, Thr268, Leu274, Asp325, Glu326, Gln329, Lys331 and Trp335 defined as flexible side chains. A grid box around the catalytic triad with dimensions of 26 Å × 26 Å × 28 Å was used to cover the entire substrate binding site. The default parameters were defined during docking with an exhaustiveness of 16. Calculations were performed using the Lamarckian genetic algorithm (LGA) method. Among the 20 calculated models, the best solution was chosen on the basis of the productive interaction of the ligand with the catalytic triad. The xylooligosaccharide template was manually constructed by attaching arabinose and xylose units to the best-docked ethyl-ferulic acid using Coot8 [25].

### 2.6 Production, purification and characterization of Xyn11 and Xyn11_m1_

The native protein Xyn11, produced in *E. coli* Rosetta (Agilent Technologies, Inc., CA, USA) using the pQE80L vector, was purified as described previously [15]. Synthetic genes (codon optimized) encoding the xylanase mutant Xyn11_m1_ were purchased from Integrated DNA Technologies (IDT). The encoding plasmid pQE80L was used to transform *E. coli* XL1-Blue (Agilent Technologies, Inc., CA, USA), and the plasmid was subsequently transferred to *E. coli* Rosetta (Agilent Technologies, Inc., CA, USA) for protein production, as previously described for the wild-type protein [15]. Briefly, *E. coli* cultures (1 L) were grown in 2 × Tryptone Yeast extract medium (2xTY) (tryptone 16 g/L, yeast extract 10 g/L, NaCl 5 g/L) at 37 °C until they reached an OD_600_ of 0.6 and then induced with 1 mM isopropyl β-D-1-thiogalactopyranoside (IPTG) (Thermo Fisher Scientific, MA, USA) at 37 °C for 5 h. The cells were subsequently harvested and disrupted by sonication, after which the resulting cell-free protein extracts were obtained. The protein variant was then purified by heat treatment at 90 °C for 10 min, followed by nickel affinity chromatography using a 1 mL HisTrap FF column (GE Healthcare, Madrid, Spain) mounted in an ÄKTA-Purifier system (GE Healthcare, Madrid, Spain), as described previously [14]. Eluted fractions displaying xylanase activity were dialyzed against 20 mM Tris-HCl, pH 7.0, containing 50 mM NaCl. The protein concentration was determined using the Bradford assay [27] (Bio-Rad Protein Assay Dye Reagent Concentrate; Bio-Rad, Madrid, Spain), with bovine serum albumin as the standard. Approximately 25 mg of protein per liter of culture was obtained, and the samples were stored at a concentration of approximately 3.2 mg/mL.

The assay conditions for xylanase activity were described previously [15]. In brief, a 1 % (w/v) oat spelt xylan or 1 % (w/v) wheat bran suspension was prepared in 20 mM Tris-HCl and 50 mM NaCl (pH 8.0). To prepare the xylan suspension, 60 mL of buffer was heated to 60 °C under stirring, followed by the addition of 1 g of oat spelt xylan (Merck Life Science S.L.U., Madrid, Spain) or 1 g of wheat bran (from a commercial source, Kellogg's, Madrid, Spain). The mixture was boiled, stirred overnight while cooling, and subsequently adjusted to 60 mL with Milli-Q water to compensate for evaporation. An additional 40 mL of buffer was then added to reach the final volume (100 mL). This treatment was intended only to homogenize the substrates, as both are poorly soluble. Importantly, this treatment did not lead to degradation because pretreated xylan without enzymes showed neither dinitrosalicylic acid (DNS) activity [15] nor detectable products (including xylose, xylooligosaccharides, and ferulic acid) with high-performance liquid chromatography (HPLC). The resulting suspension was then divided into aliquots and stored frozen until use, as xylan may degrade if kept in the refrigerator because of contaminant fungal activity. For the xylanase assays, 20 µL of Xyn11 or Xyn11_m1_ (1:300 dilution in 20 mM Tris-HCl, 50 mM NaCl, pH 8.0, from a stock solution of 3.2 mg/mL; corresponding to a final enzyme concentration of ∼1.1 µg/mL in the reaction mixture) was mixed with 180 µL of oat spelt xylan or wheat bran solution (prepared in 20 mM Tris-HCl, 50 mM NaCl, pH 8.0) and incubated for 10 min at 90 °C. Reactions were performed in sterile 2-mL safe-lock Eppendorf® polypropylene tubes (Eppendorf SE, Hamburg, Germany) with agitation (1000 rpm) in a thermoshaker (Stuart orbital incubator SI500, Stuart Scientific Co. Ltd., Staffordshire, UK). The level of degradation was monitored by quantifying the concentration of reducing sugars released using the DNS method adapted to a 96-well format [28]. The absorbance at 540 nm was measured with a BioTek Synergy H1 Multimode Reader (Agilent Technologies, Inc., CA, USA) using U-bottom 96-well microplates (Greiner Bio-One International GmbH, Kremsmünster, Austria). Additionally, the ability of the enzyme to release ferulic acid was evaluated in the same set of samples. After enzymatic inactivation by the addition of 100 µL of ethanol, the reactions were incubated at 65 °C for 10 min and then stopped on ice, under which the enzyme was completely inactive [15]. Then, the samples were subsequently centrifuged for 3 min at 13,200 rpm. The supernatants were then collected, filtered through 0.45 µm and 25 mm diameter nylon filters (Membrane Solutions, WA, USA), and analyzed with HPLC as described below. In all the cases, all the reactions were conducted in triplicate (*n* = 3), with appropriate control reactions and background signals considered.

The hydrolysis of methyl ferulate (Merck Life Science S.L.U., Madrid, Spain) was assessed using a pH indicator-based assay performed at 90 °C and a pH of 8.0 in 384-well microplates (Greiner Bio-One GmbH, Kremsmünster, Austria). The reaction was monitored by measuring the absorbance at 550 nm over a period of 0.5–1.0 min on the basis of the color change of the pH indicator phenol red (ε = 8450 M⁻¹ cm⁻¹), as previously described [13]. The acid generated by ester bond cleavage catalyzed by the hydrolytic enzyme triggered a color shift. The reaction conditions were as follows: a final volume of 44 μL in 5 mM N-(2-hydroxyethyl)piperazine-N′-(3-propanesulfonic acid) (EPPS; Merck Life Science S.L.U., Madrid, Spain) buffer (pH 8.0) supplemented with 50 mM NaCl and 0.45 mM Phenol Red® (Merck Life Science S.L.U., Madrid, Spain); an enzyme concentration of 1.0 μg/mL (Xyn11 or Xyn11_m1_); and a substrate concentration ranging from 0 to 25 mM, prepared from a 100 mg/mL stock solution in dimethyl sulfoxide (DMSO). The enzymatic activity was calculated by determining the change in absorbance at 550 nm per minute with a BioTek Synergy H1 Multimode Reader (Agilent Technologies, Inc., CA, USA) on the basis of the slope of the linear phase of the reaction, as previously described [13]. All reactions were conducted in triplicate (*n* = 3), with appropriate control reactions and background signals considered.

NaCl (sodium chloride) was included in all purification, storage, and reaction buffers to enhance protein stability and solubility. The presence of NaCl increases the ionic strength of the buffer, thereby reducing nonspecific electrostatic interactions that can lead to protein aggregation or precipitation.

### 2.7 General procedure for analytical HPLC

The reaction mixtures were analyzed with reversed-phase chromatography using a quaternary pump (Model 1100, Agilent Technologies, Inc., CA, USA) coupled to a Phenomenex Zorbax Eclipse plus C18 column (4.6 mm diameter by 100 mm length, 3.5 μm particle size; Agilent Technologies, Inc., CA, USA), with an autosampler (Model L-2200; Hitachi High-Tec., Tokyo, Japan,) and a photodiode array detector (PDA, Varian Prostar). The column temperature was maintained at 40 °C, and the flow rate was 0.8 mL/min. Each injection had a volume of 10 μL, and the analytes were eluted with a gradient of acetonitrile (CH_3_CN) and H_2_O (with 0.1 % vol/vol formic acid in both solvents), starting with 20 % CH_3_CN and 80 % H_2_O, followed by a 4 min linear gradient from 20 % CH_3_CN to 50 %, which was maintained for one minute. Afterward, the mixture was returned to the initial conditions (20:80) for 9 min. The UV detection wavelength was 322 nm. Integration of the peaks was performed using the Varian Star LC workstation 6.41 (Agilent Technologies, Inc., CA, USA). The samples were diluted 1:20 with ethanol and filtered through 0.45 µm and 25 mm diameter nylon filters (Membrane Solutions, WA, USA). A calibration curve of ferulic acid was constructed between 0 and 500 ppm.

### 2.8 Codes and accession numbers

The sequence encoding Xyn11_m1_ was deposited in UniProtKB under the accession number F7YXD6. The atomic coordinates and structural factors for the Xyn11_m1_ structure were deposited in the Research Collaboratory for Structural Bioinformatics (RCSB) Protein Data Bank with the accession code 8BBI.

## 3 Results and discussion

### 3.1 Computational search and design of a new active site in Xyn11

**Fig. 1:**
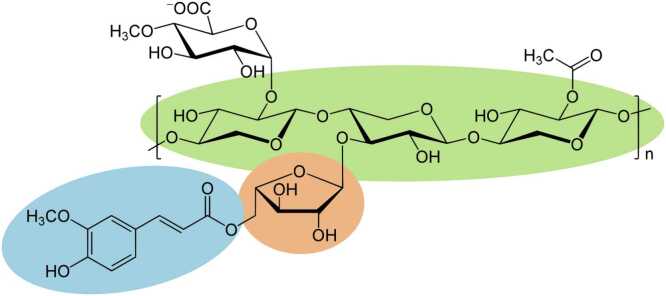
Schematic representation of the xylan polymer with ramifications. The xylan backbone is shown in green, the arabinose substitution in orange, and the ferulic acid group in blue.

We constructed a secondary catalytic site in the structure of the extremophilic xylanase Xyn11 from *Pseudothermotoga thermarum* [15] to confer esterase activity on this enzyme. Specifically, the presence of a Ser–His–Asp/Glu catalytic triad in a suitable conformation and location should endow the enzyme with feruloyl esterase debranching activity, facilitating the action of the xylanase active site on the polymer and enhancing wild-type activity (Fig. 1).

Although details are provided below, the artificial active site was introduced on the basis of specific criteria, including (i) the presence of binding pockets located at least 20 Å away from the native catalytic residues of the endoxylanase and (ii) the ability, following the incorporation of artificial Ser–His–Asp/Glu catalytic triads, to maintain catalytically competent geometries (interatomic distances < 5.0 Å), with the triad remaining in a catalytically active conformation up to 15 % of the simulation time.

**Fig. 2:**
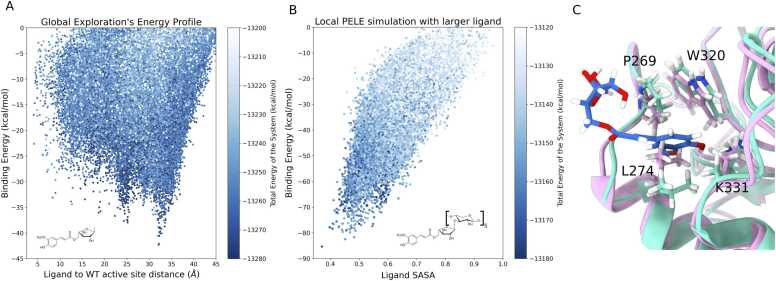
Computational analysis of the ligand binding site. (A) Interaction energy profile of global PELE exploration using feruloyl-L-arabinose. The distance from the ligand ester carbon to the nearest catalytic residue (E144 and E251) was calculated. (B) Ligand SASA (solvent accessible surface area) vs. binding energy for the local PELE simulation using feruloyl-L-arabinose bound to a 5-unit xylan polymer. This simulation focused on the local region of the identified binding site, in contrast to global exploration, which involved sampling the entire enzyme surface. (C) Comparison between the crystallographic conformation (pink, PDB code 7NL2) and the transient binding pocket identified by the PELE simulation (green). Structural visualization of the ligand–protein complex highlights the amino acid residues involved in ligand interactions: hydrophobic contacts (P269, L274, W320) and a hydrogen bond interaction (K331).

The initial step in designing a PluriZyme is to identify a surface cavity on the protein that can accommodate a specific substrate. This is usually achieved through a global exploration simulation, in which the substrate explores the entire protein surface using the site finder method within the PELE software. Fig. 2A presents the energy profile of feruloyl-L-arabinose (Supplementary Fig. 1 A) during protein global exploration. Here, we observe a significant binding energy minimum within 30–35 Å of the wild-type catalytic residues (E144 and E251). Such local minima suggest the presence of a metastable state, where the ligand is stabilized by local interactions, often indicative of a cleft or small opening on the protein surface. At this minimum, the ferulic acid moiety of the ligand occupies a transient cavity on the protein surface that is absent in the crystal structure.

Following the identification of the pocket, a local PELE simulation was conducted using a larger, more chemically relevant ligand, a 5-unit xylan polymer (Supplementary Fig. 1B). The energy profile from this simulation is shown in Fig. 2B. In the interaction energy minima, the carbonyl carbon of the ester bond in the ligand is positioned near several side chains at the top of the cavity. This configuration provides a promising starting point for further engineering to introduce catalytic residues aimed at esterase activity.

Notably, this cavity emerges owing to a conformational change in a leucine residue (L274), which undergoes a significant shift during the simulation to create an accessible binding site. Moreover, other amino acid side chains have torsions to accommodate the ligand (K331, V256). The interaction between the ferulic acid ligand and the residues lining this transient cavity is shown in Fig. 2C. The figure reveals specific molecular contacts, mainly hydrophobic interactions facilitated by the repositioned leucine, that contribute to the stability of this pose. Comparable mechanisms have been observed in naturally evolved enzymes, where substrate binding often relies on induced fit or conformational changes [29], [30]. These observations underscore the importance of transient protein dynamics in identifying new binding sites that could be exploited for enzyme design or even functional modulation and that might not be captured in static crystallographic models. In Fig. 2C, the superposition of the crystallographic structure (PDB 7NL2) and the pose from the PELE simulation with the interacting residues is shown.

**Fig. 3:**
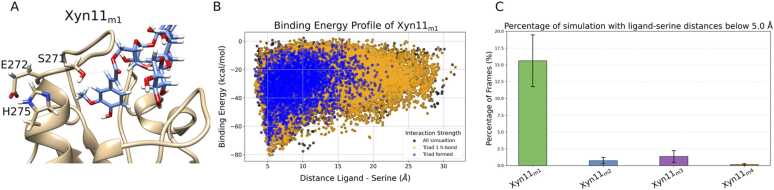
Computational analysis of ligand binding and active site engineering. (A) Structural visualization of Xyn11_m1_ (L271S, K275H and E272 (WT)) interacting with the ligand in a catalytic pose. (B) Binding energy profiles for Xyn11_m1_, showing the ligand–serine distance vs. the interaction energy between the ligand and the engineered active site. Color-coded representations of the catalytic residue interactions are shown: black (all simulation frames), orange (simulation frames where at least one triad distance is satisfied), and blue (the two catalytic triad distances are satisfied). (C) Results from molecular dynamics simulations (8 replicas, 200 ns each) illustrating the stabilization of the ligand in a catalytic conformation within the engineered active sites. The percentage of poses in which the ligand achieves catalytic alignment is shown for all the tested mutants.

The second step in PluriZyme design centers on introducing a catalytic triad, consisting of serine, histidine, aspartate or glutamate, to confer esterase activity to the enzyme. The design process involved the manual insertion of mutations on the basis of structural modeling and visual inspection, ensuring that the catalytic residues were correctly oriented relative to the substrate. This careful placement was critical to achieve optimal catalytic distances during the PELE and MD simulations of the substrate–enzyme complex. These distances include two within the triad (as hydrogen bonds must be maintained) and one between the serine and the ester bond of the substrate. The inserted triads were Xyn11_m1_ (L271S, K275H and E272 (wild type, WT)), Xyn11_m2_ (L271S, L274H, and K275D), Xyn11_m3_ (K331S, Q329H, and E326 (WT)), and Xyn11_m4_ (K331S, D325H, and Q329D). The triad 1 conformation is shown in Fig. 3A.

To assess the impact of these engineered catalytic triads, local induced-fit PELE simulations were performed using the ferulic-xylan ligand (Supplementary Fig. 1B). These simulations allowed us to compare the interaction energy profiles of the mutant designs, with a focus on the spatial proximity between the catalytic serine and the ligand's ester bond. In addition, they facilitated the identification of energetically favorable configurations and provided a quantitative assessment of the binding efficiency of the mutants. One design, Xyn11_m1_, exhibited favorable energy minima and a high frequency of catalytic poses at the targeted active site (Fig. 3B), whereas the other three did not show a significant energy minimum when the ligand was close to the active site or when the catalytic triad was not stabilized (Supplementary Fig. 2). Moreover, to further validate the performance of the engineered triads, MD simulations were conducted. These simulations, comprising 8 replicate runs of 200 ns each, evaluated the stability of the ligand within the newly created active sites. The results demonstrated that the Xyn11_m1_ mutant consistently stabilized the ligand in a catalytic conformation (Fig. 3C). This stability underscores the feasibility of the introduced catalytic activity and reinforces the potential of the engineered PluriZyme to exhibit effective esterase functionality.

### 3.2 *Functional characterization of Xyn11*_*m1*_

Before describing the catalytic capacities of the engineered construct, it is important to clarify the rationale behind the assay conditions employed. The optimal conditions for the wild-type enzyme were previously established: maximal xylanolytic activity at 90 °C and an alkaline pH of 10.5, with negligible activity below 70 °C [15]. Accordingly, all the assays with Xyn11 and Xyn11_m1_ were performed at 90 °C, the optimal temperature for the scaffold. For the evaluation of feruloyl esterase activity, however, the pH was set at 8.0 rather than 10.5. This pH value was chosen because methyl ferulate is unstable at high pH under high temperature, and the colorimetric assay employed (Phenol Red–based) performs optimally at pH 8.0 [13]. For this reason, the assays with xylans were also conducted at this pH. We did not perform a full characterization of the optimal conditions for the newly introduced esterase site, as xylanase activity is negligible below 70 °C and the PluriZyme construct already operates efficiently at 90 °C.

Xyn11 exhibited specific activity toward oat spelt xylan, with approximately 600 ± 8 µmol of reducing sugars (expressed as xylose equivalents)/min/mg of enzyme (Table 2; [14]). We found that the xylanase activity of Xyn11_m1_ was similar to that of Xyn11, with 713 ± 4 µmol reducing sugars (xylose)/min/mg protein (Table 2). In all the cases, the oat spelt xylan was pretreated to homogenize the substrate and improve its solubility before the tests (see 2.6). The comparable activity levels of Xyn11_m1_ and Xyn11 can be attributed to the use of oat spelt xylan, which is typically employed as a model substrate for xylanases but does not contain ferulic acid [15]. The small increase in activity in other PluriZymes has been linked to the fact that the introduction of mutations to incorporate a new active site may have the potential to impact stability, exerting both local and global effects as a result of an additional hydrogen bond pattern [11]. These new hydrogen bonds inserted along the triad (serine–histidine and histidine–acid–hydrogen bonds) may contribute to local stabilization around the engineered site or reinforce existing structural elements, thereby subtly increasing overall enzyme activity. The esterase activity was further assessed with a pH indicator assay [13] at 90 °C and a pH of 8.0 using methyl ferulate as a model ferulic ester. Under the assay conditions, Xyn11_m1_ exhibited esterase activity toward the synthetic substrate methyl ferulate, a commonly used model compound for assessing feruloyl esterase activity, with a *K*_m_ of 2.9 ± 0.2 mM and a V_max_ of 140 ± 5 µmol/min/mg protein (Supplementary Fig. 3). This activity was not observed in the native protein, indicating that esterase function was acquired upon engineering.

**Table 2:** Hydrolytic activity of Xyn11 and Xyn11_m1_.

Variant	Xylanase activity (µmol/min/mg)^a^		Feruloyl esterase activity (µmol/min/g)^a^
	Oat spelt xylan	Wheat bran xylan	Methyl ferulate
Xyn11	600 ± 8	222 ± 9	n.d.
Xyn11_m1_	713 ± 4	513 ± 27	140 ± 5

a Measured at 90 °C, pH 8.0.

When engineering or designing a new active site on the basis of computer simulations, a key question is whether the resulting constructs can achieve catalytic performance levels comparable to those of natural enzymes. Recent studies have demonstrated that it is possible to generate enzyme scaffolds with artificial active sites that approach native catalytic efficiency [12], [13], [31], [32]. The findings of the present study support this notion, showing that the introduction of an artificial catalytic triad, comprising Ser–His–Asp/Glu, into Xyn11 enabled the creation of a dual xylanase–feruloyl esterase scaffold, with feruloyl esterase activity comparable to that of natural feruloyl esterases, including those derived from thermophilic organisms (Supplementary Table 1).

Given that oat spelt xylan, which does not contain ferulic acid, was used, we could not evaluate the advantages of incorporating secondary feruloyl esterase activity into the debranching activity of xylans that contain feruloyl moieties in their structure. For this reason, we selected wheat bran xylan, a rich source of phenolic acids, especially ferulic acid, which is primarily bound to arabinoxylans in the cell wall of plant cells. Studies have reported ferulic acid concentrations in wheat bran ranging from 12 to 4527 µg/g, depending on the fraction analyzed and the extraction conditions used [33], [34], [35], [36], [37]. Because species-related differences in the feruloylated side-chain profiles of grain arabinoxylans have been observed, leading to differences in arabinoxylan functionality and resistance [38], and no reference material is commercially available, we used wheat bran from a commercial source (Kellogg’s, Madrid, Spain), which contains 86 % wheat bran. Although the exact ferulic acid concentration in this material is unknown, the goal of our study was to demonstrate that embedding a feruloyl esterase active site into a thermophilic endoxylanase scaffold could promote the degradation of feruloylated xylans. Given that our enzymatic assays revealed the release of ferulic acid, we did not consider it necessary to perform a detailed compositional analysis. To evaluate the efficiency of wheat bran degradation by Xyn11_m1_, experimental conditions similar to those employed with oat spelt xylan were used. Wheat bran was pretreated in the same manner as oat spelt xylan to homogenize the substrate and improve its solubility. Additionally, we evaluated the capacity of the native and engineered enzymes to release ferulic acid from wheat bran using HPLC. First, we observed that Xyn11 hydrolyzes wheat bran xylan, although its specific activity is approximately 2.7 times lower than that observed for oat spelt xylan (Table 2), possibly owing to structural and functional differences between the two xylans. Second, compared with the native enzyme, Xyn11_m1_ exhibited approximately 2.3-fold greater xylanase activity toward wheat bran xylan. In contrast, no significant difference in activity was observed when oat spelt xylan was used as the substrate, with both enzymes showing comparable levels of activity (Table 2). HPLC analysis further confirmed the release of ferulic acid when wheat bran xylan was treated with Xyn11_m1_, a phenomenon not observed with Xyn11 (Supplementary Fig. 4). These results suggest that the higher degradation rate of this recalcitrant polysaccharide in the presence of Xyn11_m1_ may be attributable to the feruloyl esterase activity incorporated into this enzyme, which is absent in Xyn11. This activity likely facilitates the cleavage of feruloyl moieties within the feruloylated xylan polymer through the artificially introduced active site, making the xylan backbone more accessible for degradation by the native endoxylanase active site.

Comparable strategies to enhance the degradation of feruloylated xylans have been described in the literature and are typically based on enzyme mixtures or engineered multifunctional catalysts. In this regard, the higher hydrolytic efficiency observed for Xyn11_m__1_ in hydrolyzing wheat bran than for Xyn11 resembles the performance of other multifunctional systems in which feruloyl esterase and endoxylanase activities act in concert. For instance, combinations of feruloyl esterases and xylanases, or the cocultivation of feruloyl esterase- and xylanase-producing strains, have been reported to efficiently release ferulic acid from wheat bran alone [35] or from lignocellulose derived from distillers’ grains, with the solid residues remaining after the distillation of fermented grains, mainly composed of wheat bran and sorghum [39]. However, such release did not always correlate with an increased production of reducing sugars, suggesting that ferulic acid removal is not invariably linked to enhanced xylan hydrolysis. In contrast, other studies have demonstrated that the presence of a feruloyl esterase can substantially increase xylan backbone hydrolysis, as exemplified by the combination of a feruloyl esterase with a GH10 endoxylanase on corn fiber (pH 7.0, 40 °C, 2 h), which led to a fourfold improvement in hydrolysis of the main xylan chain [40].

The PluriZyme Xyn11_m1_ designed in this work also represents an additional example of a multifunctional biocatalyst for polysaccharide degradation, comparable to previously reported naturally occurring bifunctional enzymes [41] and chimeric proteins harboring multiple catalytic domains. With respect to xylan degradation, relevant cases include chimeras obtained by fusing two xylanases [42]; a xylanase with a feruloyl esterase [41], [43], [44]; a xylanase, a glucanase and a feruloyl esterase [45]; or a β-glucosidase, a xylanase and a feruloyl esterase [46]. For example, compared with the parental Xyn11, PluriZyme Xyn11_m1_ clearly improved the hydrolysis of the xylan backbone, producing ∼2.3-fold more reducing sugars under harsh conditions (90 °C, pH 7.0, 10 min). Such an enhancement is in line with the results obtained by Wang et al. [44] who used an engineered bifunctional enzyme (XynII-Fae) created by fusing the feruloyl esterase domain from a natural bifunctional Xyn-Fae with the β-xylanase XynII from *Trichoderma reesei*. In this case, hydrolysis of delignified corn yielded 2.43 mg/mL reducing sugars with XynII-Fae compared with 0.98 mg/mL with XynII alone, corresponding to an ∼2.5-fold increase, closely mirroring the improvement observed with Xyn11_m1_. However, the naturally occurring bifunctional xylanase/feruloyl esterase rXyn10A/Fae1A described by Wang et al. [41] produced ferulic acid yields ranging from 1.15 to 7.31 mg/g from diverse substrates, including soluble wheat arabinoxylan, destarched wheat bran, ultrafine-grinded corn stover, and steam-exploded corncob (40 °C, pH 6.0, 24 h, 200 rpm). However, the presence of the feruloyl esterase domain did not significantly affect reducing sugar production, suggesting that the contribution of feruloyl acid release to xylan backbone hydrolysis was limited. These observations highlight that the extent to which ferulic acid release contributes to the generation of xylanase-derived products strongly depends on the specific enzymes employed and their catalytic efficiency.

Taken together, these comparisons demonstrate that Xyn11_m1_ not only expands the catalytic repertoire of xylanases but also exemplifies the broader potential of PluriZymes as rationally designed, multifunctional catalysts capable of competing with both enzyme cocktails and fusion proteins for the efficient degradation of recalcitrant polysaccharides, herein exemplified by feruloylated xylans.

### 3.3 Folding of Xyn11_m1_

**Fig. 4:**
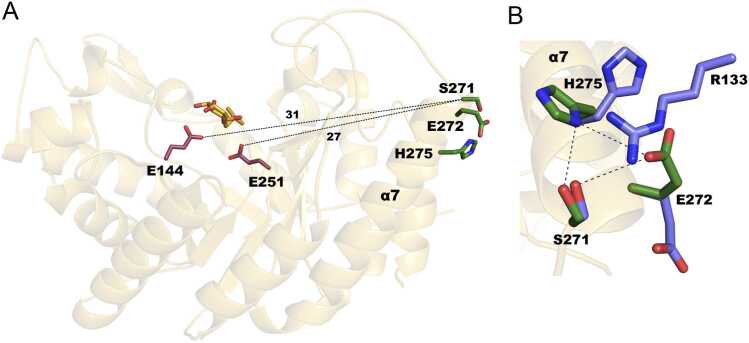
**Structural insights into Xyn11**_**m1**_**.** (A) Xyn11_m1_ folding. The intrinsic xylanase catalytic pair (E144 and E251) is shown in stick representation in raspberry, whereas the artificial secondary esterase triad (S271, E272 and H275) is shown in stick representation in green. The trapped IPTG molecule is shown in stick representation in orange. (B) Superimposition of the two independent molecules within the asymmetric unit, chain A (blue) and chain B (green), at the artificial esterase secondary catalytic site. The catalytic triad of molecule B shows the hydrogen bonding pattern that is conserved among esterases.

The crystal structure of the double mutant corresponding to Xyn11_m1_ was obtained with X-ray diffraction at 2.1 Å (Table 1). The TIM barrel architecture is typical of GH10 xylanases. The intrinsic xylanase active site, located at the axis of the barrel, is formed by the residue pair E144 and E251 and includes a trapped IPTG molecule. The second artificial catalytic triad is formed by residues S271, E272 and H275 at the beginning of α7 (Fig. 4A). As in the native crystal, the double mutant Xyn11_m1_ contains two molecules in the asymmetric unit that present some conformational differences at the artificial esterase catalytic triad. Only chain B presents the S271–E272–H275 triad in a conformation that displays the hydrogen bonding pattern conserved in reported esterases (Fig. 4B). In chain A, R133, present in an adjacent chain as a result of symmetry-related crystallographic conditions, forms a hydrogen bond with S271, resulting in a shift in the E272 and H275 side chains (Fig. 4B). Such a symmetry-related contact, however, should not be present in solution.

No significant structural differences were revealed by superimposition of the wild-type and variant coordinates. As expected, in the absence of ligand, no signs of the cryptic pocket were observed in our Monte Carlo-induced fit simulations. Attempts to crystallize Xyn11_m1_ with different irreversible inhibitors were made, but the ligand was not captured in the crystal. An additional quick test using side chain flexible docking was performed using the coordinates of chain B, which presents an active conformation of its catalytic triad. Although some docked solutions (Supplementary Fig. 5 A) exhibit productive interactions with the catalytic triad, pocket opening is not accomplished with docking techniques. The ligand, however, adopts the tetrahedral conformation typical of the intermediate of ester hydrolases, where the nucleophilic serine is located under the carbonyl group to enable attack, with the oxygen being close to two glycines that stabilize the formed oxoanion [47]. Thus, one could hypothesize that multiple catalytic poses exist. Furthermore, arabinose, xylose, and ferulic acid units can be manually attached to the ligand to illustrate the putative binding of xylan (Supplementary Fig. 5B). As shown in the figure, the polymeric xylose chain extends far from the protein surface and therefore has no impediment to the insertion of its terminal ferulic moieties into the artificial esterase active site of Xyn11_m1_.

## 4 Conclusions

In this work, we successfully engineered a multifunctional enzyme, Xyn11_m1_, by introducing a feruloyl esterase active site into the Xyn11 thermophilic xylanase scaffold using the computationally guided PluriZyme approach. The new esterase site was designed using PELE simulations and molecular dynamics, resulting in a stable serine–histidine–glutamate catalytic triad on the endoxylanase Xyn11 surface without compromising the intrinsic xylanase activity. Xyn11_m1_ exhibited dual functionality, retaining xylanase activity comparable to that of the wild-type enzyme on oat spelt xylan and acquiring feruloyl esterase activity, as evidenced by its ability to hydrolyze methyl ferulate and release ferulic acid from wheat bran xylan, which subsequently resulted in a 2.3-fold improvement in hydrolysis of the main xylan chain of feruloylated xylans after deferruloylation by the introduction of feruloyl esterase activity. This highlights the synergistic advantage of combining feruloyl-debranching and xylan-backbone hydrolysis in a single-enzyme scaffold.

This study demonstrates the potential of the PluriZyme concept to expand enzymatic functionalities and provides a basis for efficient strategies in the bioconversion of complex plant biomass. Specifically, Xyn11_m1_ not only broadens the catalytic repertoire of xylanases but also exemplifies the broader promise of PluriZymes as rationally designed, multifunctional catalysts capable of competing with both enzyme cocktails, naturally occurring bifunctional enzymes and multifunctional fusion proteins or chimeras for the efficient degradation of recalcitrant polysaccharides, as exemplified here by feruloylated xylans.

All these systems have their advantages and limitations. For example, bifunctional enzymes, either naturally occurring or engineered multifunctional chimera constructs, often display intramolecular synergism during biomass degradation and can outperform enzyme mixtures in viscous and crowded environments [48]. In such cases, intermediates can be directly transferred between catalytic sites within the same protein rather than diffusing into solution, thereby improving catalytic efficiency and reducing reaction times. Compared with enzyme cocktails, fusion enzymes also benefit from simpler and more cost-effective production. Nonetheless, their performance can be compromised by challenges such as protein misfolding, structural interference, or instability of the linkers connecting catalytic domains. In contrast, PluriZymes provide an alternative strategy by embedding multiple active sites directly into a single protein scaffold. This approach circumvents the need for artificial linkers and can even enhance protein stability through novel intramolecular interactions [12]. Importantly, relying on a single protein with multifunctionality is advantageous under industrial catalytic conditions, which are often harsh and make it difficult to identify pairs of enzymes with comparable stabilities. Instead, if one enzyme has already been optimized for industrial use, small modifications to introduce a second active site, as in PluriZymes, typically do not alter its physicochemical properties. Moreover, PluriZymes allow for the rational design of entirely new catalytic functions ad hoc [12], [13], as illustrated here by the introduction of feruloyl esterase activity into Xyn11_m1_, a functionality absent from its wild-type counterpart.

It remains to be evaluated whether our proof-of-concept study could be extended to other enzymes involved in the hydrolysis of complex polysaccharides, such as cellulases or additional hemicellulases, as well as by incorporating accessory activities such as acetyl esterase, glucanase, or glucuronidase, for the development of effective biocatalysts for biomass degradation beyond the feruloylated xylans tested in this study. The application of the enzymatic scaffold with dual endoxylanase–feruloyl esterase activity designed here to real systems based on the hydrolysis of feruloylated xylans will, in the future, enable us to assess its versatility for improving lignocellulosic biomass degradation and generating value-added products such as ferulic acid and antioxidant oligosaccharides. At the methodological level, the idea of introducing artificial feruloyl esterase activity into a native endoxylanase scaffold is particularly interesting for several reasons. Although previous studies have demonstrated that it is possible to engineer artificial active sites into noncatalytic proteins or existing enzymes, most of the target scaffolds reported to date are mesophilic. Extending the PluriZyme concept, as herein reported, to thermophilic proteins, which are characterized by distinct profiles of rigidity and flexibility compared with their mesophilic counterparts, demonstrates the versatility of this approach and its applicability to structurally diverse proteins operating under different thermal conditions.

## CRediT authorship contribution statement

**Fadia V. Cervantes:** Writing – review & editing, Validation, Methodology, Formal analysis. **Jose L. Gonzalez-Alfonso:** Writing – review & editing, Validation, Resources, Methodology, Formal analysis. **Julia Sanz-Aparicio:** Writing – review & editing, Writing – original draft, Visualization, Validation, Supervision, Resources, Project administration, Methodology, Investigation, Funding acquisition. **Manuel Ferrer:** Writing – review & editing, Writing – original draft, Supervision, Resources, Project administration, Methodology, Investigation, Funding acquisition, Conceptualization. **Francisco J. Plou:** Writing – review & editing, Supervision, Project administration, Methodology, Funding acquisition. **Julio Polaina:** Writing – review & editing, Supervision, Methodology, Investigation, Funding acquisition, Conceptualization. **Rubén Muñoz-Tafalla:** Writing – review & editing, Writing – original draft, Visualization, Software, Resources, Methodology, Investigation, Formal analysis, Data curation. **Víctor Guallar:** Writing – review & editing, Writing – original draft, Supervision, Software, Resources, Project administration, Methodology, Investigation, Funding acquisition, Conceptualization. **David Talens-Perales:** Writing – review & editing, Writing – original draft, Validation, Resources, Project administration, Methodology, Investigation, Conceptualization. **Isabel Cea-Rama:** Visualization, Validation, Methodology, Investigation, Formal analysis, Data curation.

## Declaration of Generative AI and AI-assisted technologies in the writing process

During the preparation of this work, the author(s) did not use generative AI or AI-assisted technologies for content creation. Language editing was limited to grammar and spelling checks provided by a professional editing service (American Journal Experts), which does not involve the use of AI.

## Funding statement

The authors (M.F., V.G.) thank the 10.13039/501100000780European Union’s Horizon 2020 Research and Innovation Programme for grant 101000327-FuturEnzyme and Horizon Europe for grant 101060625-Nymphe (M.F.). They also acknowledge funding from the Ministerio de Ciencia, Innovación y Universidades, Agencia Estatal de Investigación (AEI) (MICIU/AEI/10.13039/501100011033), FEDER, the EU, and the European Union NextGenerationEU/PRTR, supporting projects PID2020-112758RB-I00 (M.F.), PDC2021-121534-I00 (M.F.), TED2021-130544B-I00 (M.F.), PID2023-153370OB-I00 (M.F.), PID2019-106370RB-I00 (V.G.), and PID2022–136367OB-C31/C33 (F.J.P. and J.S.). We thank the Synchrotron Radiation Source at ALBA (Barcelona, Spain) for assisting with the BL13-XALOC beamline.

## Declaration of Competing Interest

There are no known conflicts of interest associated with this publication.

## Data Availability

The PELE and Molecular Dynamics simulations have been deposited at Zenodo under the identifier https://doi.org/10.5281/zenodo.15601971. To use the archive, download the file, and extract its contents to a local directory using appropriate software. The directory contains separate folders for each type of simulation, along with input, output and README files. The Xyn11_m1_ sequence (UniProtKB: F7YXD6) and structure (PDB: 8BBI) have been deposited in public databases.

## Footnotes

Appendix A Supplementary data associated with this article can be found in the online version at doi:10.1016/j.csbj.2025.09.003.
