# Supplementary material for: Embedding a feruloyl esterase active site into a thermophilic endoxylanase scaffold for the degradation of feruloylated xylans
Source: Comput Struct Biotechnol J. 2025 Sep 3;27:3814–23. doi: 10.1016/j.csbj.2025.09.003 (PMC12454871; doi:10.1016/j.csbj.2025.09.003)
Supplement: Supplementary file 1 — Supplementary material [file mmc1.docx]

**Supplementary material**

Embedding a Feruloyl Esterase Active Site into a Thermophilic Endoxylanase Scaffold for the Degradation of Feruloylated Xylans

Rubén Muñoz-Tafalla^1,2^, Isabel Cea-Rama^3^, Fadia V. Cervantes^4^, Jose L. Gonzalez-Alfonso^4^, Francisco J. Plou^4^, Julio Polaina^5^, Julia Sanz-Aparicio^3,*^, Manuel Ferrer^4,*^, Víctor Guallar^1,6,*^ David Talens-Perales^5,*^

**Appendix**

Supplementary Table 1…………………………………………………………………………………..2

Supplementary Figure 1………………………………………………………………………………….3

Supplementary Figure 2………………………………………………………………………………….4

Supplementary Figure 3………………………………………………………………………………….5

Supplementary Figure 4………………………………………………………………………………….6

Supplementary Figure 5………………………………………………………………………………….7

References…………………………………………………………………………………………………8

**Supplementary Table 1.** Comparative overview of microbial feruloyl esterases. This table presents a comparative overview of various microbial feruloyl esterases, including the newly developed artificial thermostable enzyme used in this study. The data include the source organism, optimal temperature (T_opt_), specific activity against methyl ferulate (expressed in U/mg), and corresponding reference.

| Enzyme | Organism | Topt (°C) | Specific activity (U/mg)^1^ | Reference |
| --- | --- | --- | --- | --- |
| GthFAE (T41N/T150R) | *Geobacillus thermoglucosidasius* | 65 | 41.8 | [1] |
| Tx-Est1 (CE1) | *Thermobacillus xylanilyticus* | 60 | 29.2 | [2] |
| StFaeA | *Sporotrichum thermophile* | 55–60 | 10.9 | [3] |
| MtFae1a | *Myceliophthora thermophila* | 55 | 11.3 | [4] |
| PhFAE (S155F) | *Pandoraea horticolens* | 40 | 1.18 | [5] |
| DfFAE | *Dorea formicigenerans* | 40 | 2.31 | [6] |
| *C. stercorarium* FAE | *Clostridium stercorarium* | 65 | 131.0 | [7] |
| EstG34 | Metagenome (compost, thermophilic) | ~70 | 9.2 | [8] |
| FaeI | *Cellulosilyticum ruminicola* H1 | ND | 4.75 | [9] |
| BpFAE | *Bacillus pumilus* SK52.001 | 50 | 377.9 | [10] |
| PET46 | *Candidatus Bathyarchaeota* | ~75 | 45.0 | [11] |
| PET hydrolases | Diverse (bacteria and fungi) | 30–75 | NR | [12] |

^1^NR, not reported

**Supplementary Fig. 1.** Tested ligands. (A) Feruloyl-L-arabinose. (B) Feruloyl-L-arabinose bound to a 5-unit xylan polymer. Coloring match Figure 1, with the xylan backbone shown in green, the arabinose substitution in orange, and the ferulic acid group in blue.


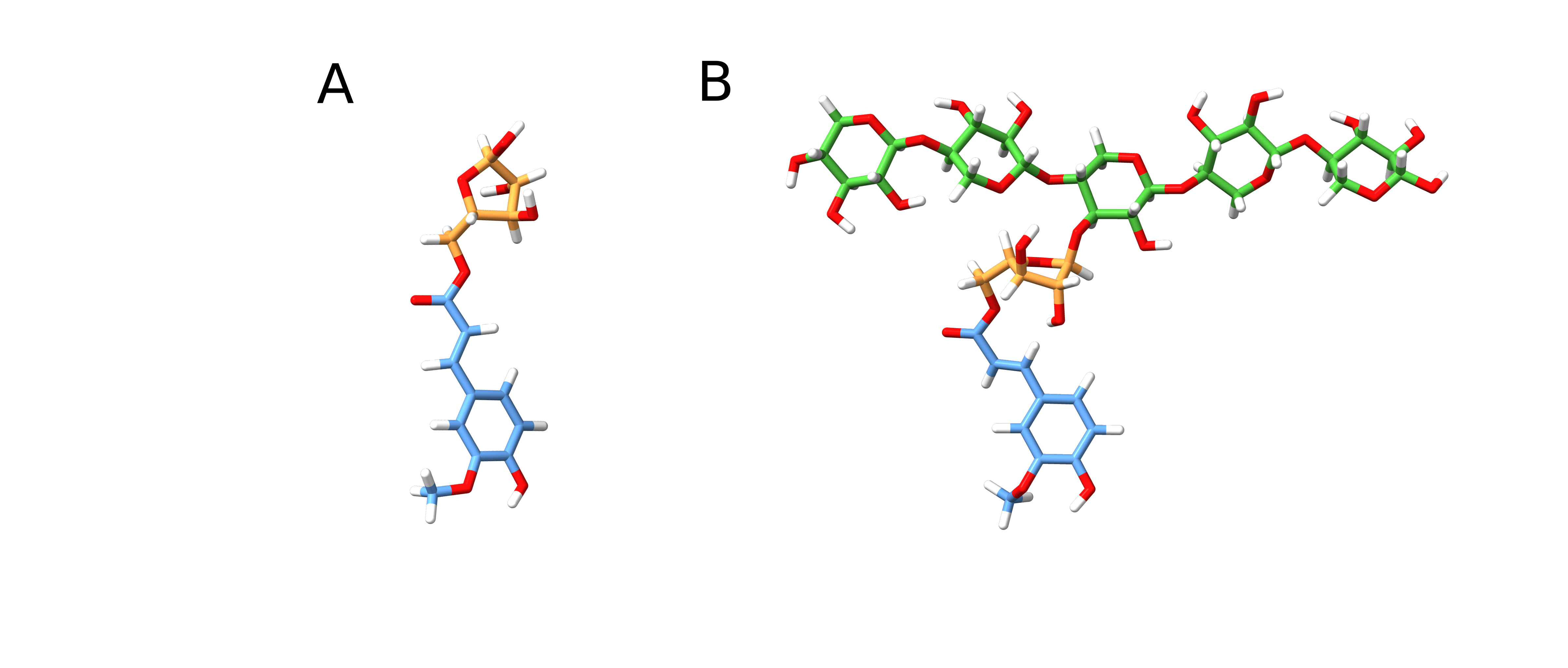


**Supplementary Fig. 2.** Interaction energy profiles for the Xyn11_m2_ (A), Xyn11_m3_ (B), and Xyn11_m4_ (C) variants. Binding energy profiles showing the ligand‒serine distance vs. the interaction energy between the ligand and the engineered active site are shown. Color-coded representations of the catalytic residue interactions are shown: black (all simulation frames), orange (simulation frames where at least one triad distance is accomplished), and blue (both catalytic triad distances are accomplished).


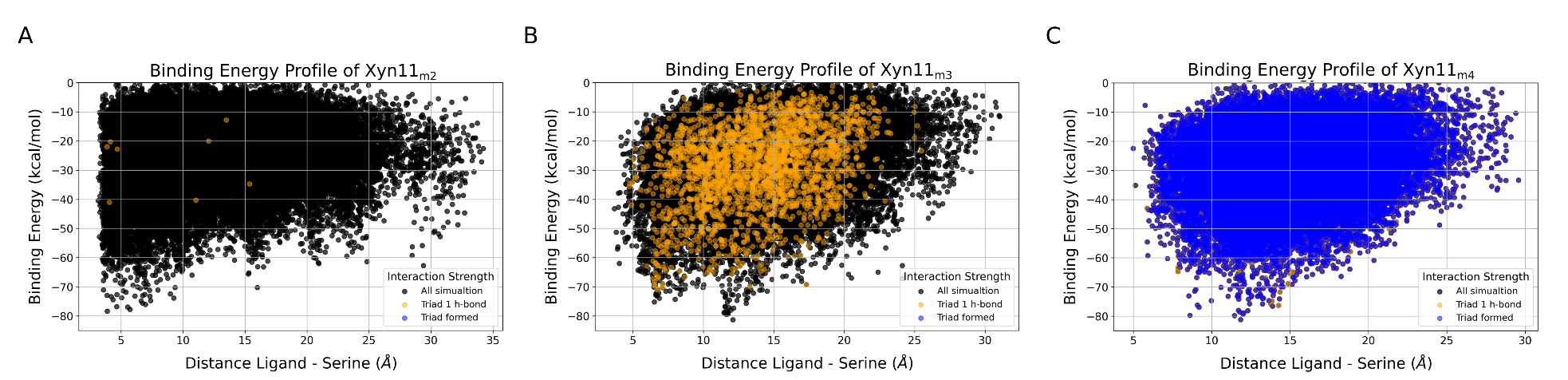


**Supplementary Fig. 3.** Specific activity of Xyn11_m1_ toward methyl ferulate at different substrate concentrations. The hydrolysis of methyl ferulate was measured at 90 °C and pH 8.0 using a pH indicator assay in 384-well plates. The reactions (44 μL) contained 1.0 μg/mL Xyn11_m1_, 0–25 mM methyl ferulate, 5 mM EPPS buffer (pH 8.0), and 0.45 mM Phenol Red^®^. The absorbance at 550 nm was continuously monitored with a Synergy HT Multi-Mode Microplate Reader, and the specific activity was calculated from the initial slopes. Assays were performed in triplicate (*n* = 3), including controls and background correction. Under these reaction conditions, Xyn11 showed no detectable activity, and the signals were similar to the background.

**Supplementary Fig. 4.** Representative HPLC chromatogram showing ferulic acid release during xylan hydrolysis of wheat bran by Xyn11_m1_. After reactions the samples (200 µL) were inactivated by adding 100 µL of ethanol and centrifuged for 3 minutes at 13200 rpm, and the supernatants were collected, filtered with 0.45 µm nylon filters and analyzed by HPLC. The Y-axis represents absorbance in arbitrary units. The “background” sample corresponds to the reaction mixture without the addition of the enzyme (dashed pink line). Under these conditions, Xyn11 showed no detectable activity, and the signals were comparable to the background.


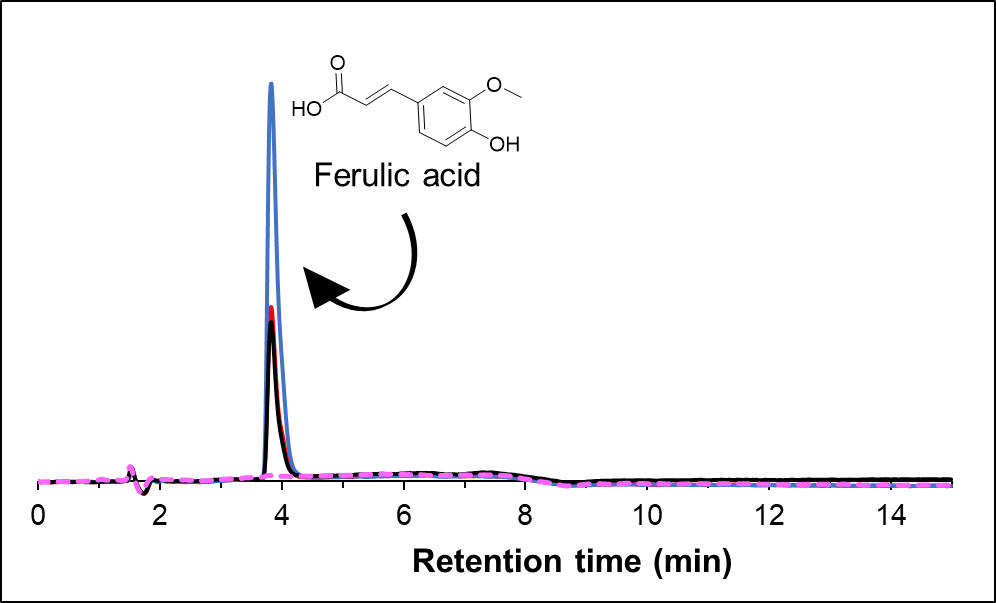


**Supplementary Fig. 5**. Docking and binding of substrates during Xyn11_m1_ conformational shifts and xylan template interactions. **(**A) Best model obtained after automatic docking of ethyl 4'-hydroxy-3'-methoxycinnamate (in violet) within the artificial esterase active site of Xyn11_m1_. The residues considered flexible are shown in stick representation (in orange), highlighting the conformational changes from the free state (in forest green). Hydrogen bonds are shown as dashes, whereas covalent bonds are shown as continuous lines. (B) Arabinose (in yellow), xylose (in pale blue) and the two ferulic acid units (in purple and marine green) units were manually built into the automatically docked substrate to illustrate the putative binding of a xylan template. The cavity of the artificial second active site is depicted in a surface representation of the protein (in orange). The catalytic triad is shown in stick representation in orange.


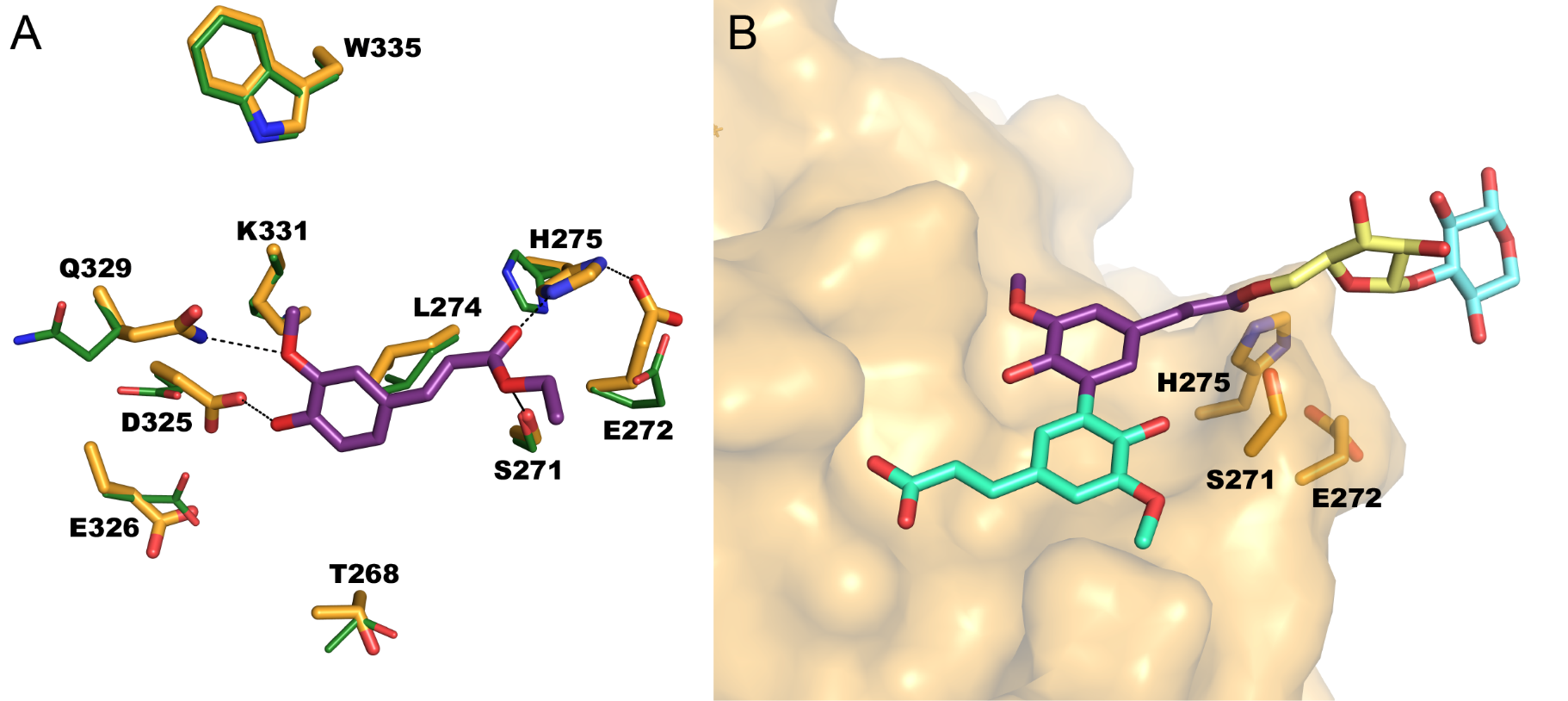


**Supplementary references**

1. Yang W, Sun L, Dong P, Chen Y, Zhang H, et al. (2022) Structure-guided rational design of the *Geobacillus thermoglucosidasius* feruloyl esterase GthFAE to improve its thermostability. Biochem Biophys Res Commun 16(600): 117–122. https://doi.org/10.1016/j.bbrc.2022.02.074
2. Garbelotti CV, Bulmer GS, Ward RJ, van Munster JM. (2023) The feruloyl esterase from *Thermobacillus xylanilyticus* shows broad specificity for processing pre-biotic feruloylated xylooligosaccharides at high temperatures. Food Chem 405: 134939. https://doi.org/10.1016/j.foodchem.2022.134939
3. **Crepin VF, Faulds CB, Connerton IF (2004)** Functional classification of the microbial feruloyl esterases. Appl Microbiol Biotechnol 63(5): 647–652. https://doi.org/10.1007/s00253-003-1476-3
4. Topakas E, Moukouli M, Dimarogona M, Christakopoulos P. (2012) Expression, characterization and structural modelling of a feruloyl esterase from the thermophilic fungus *Myceliophthora thermophila*. Appl Microbiol Biotechnol 94(2): 399–411. https://doi.org/10.1007/s00253-011-3612-9
5. **Yang S, Lin M, Chen J, Liu M, Chen Q (2025)** Engineering of an alkaline feruloyl esterase PhFAE for enhanced thermal stability and catalytic efficiency through molecular dynamics and FireProt. Catalysts 15(1) :92. <https://doi.org/10.3390/catal15010092>
6. **Yang S, Wang Y, Wei X, Wen B, Liu S, et al. (2022)** Engineering the active site pocket to enhance the catalytic efficiency of a novel feruloyl esterase derived from human intestinal bacteria *Dorea formicigenerans*. Front Bioeng Biotechnol 10: 936914. <https://doi.org/10.3389/fbioe.2022.936914>
7. **Donaghy JA, Bronnenmeier K, Soto-Kelly PF, McKay AM (2000)** Purification and characterization of an extracellular feruloyl esterase from the thermophilic anaerobe *Clostridium stercorarium*. J Appl Microbiol 88(3): 458–466. https://doi.org/10.1046/j.1365-2672.2000.00983.x
8. Ohlhoff CW, Kirby BM, Van Zyl L, Mutepfa DLR, Casanueva A, et al. (2015) An unusual feruloyl esterase belonging to family VIII esterases and displaying a broad substrate range. J Mol Catal B Enzym 118: 79-88. https://doi.org/10.1016/j.molcatb.2015.04.010
9. Topakas E, Moukouli M, Dimarogona M, Christakopoulos P (2012) Expression, characterization and structural modelling of a feruloyl esterase from the thermophilic fungus *Myceliophthora thermophile.* Appl Microbiol Biotechnol 94: 399–411. <https://doi.org/10.1007/s00253-011-3612-9>
10. Perez-Garcia P, Chow J, Costanzi E, Gurschke M, Dittrich J, et al. (2023) An archaeal lid-containing feruloyl esterase degrades polyethylene terephthalate. Commun Chem 6: 193. <https://doi.org/10.1038/s42004-023-00998-z>
11. **Zhou Y, Zhang Y, Wang Y, Wang Y, Li W, et al. (2023)** Structural basis for the catalytic mechanism of a novel PET hydrolase. Commun Chem 6: 998. <https://doi.org/10.1038/s42004-023-00998-z>
12. Wei R, Westh P, Weber G, Blank LM, Bornscheuer UT (2025) Standardization guidelines and future trends for PET hydrolase research. Nat Commun 16: 4684. <https://doi.org/10.1038/s41467-025-60016-9>
